# The Brief Suicide Cognitions Scale: Development and Clinical Application

**DOI:** 10.3389/fpsyt.2021.737393

**Published:** 2021-09-14

**Authors:** M. David Rudd, Craig J. Bryan

**Affiliations:** ^1^Department of Psychology, University of Memphis, Memphis, TN, United States; ^2^Department of Psychiatry and Behavioral Health, The Ohio State University Wexner Medical Center, Columbus, OH, United States

**Keywords:** suicide risk assessment, acute risk, Chronic risk, long-term risk, Brief Suicide Cognitions Scale

## Abstract

The study explored the development of the Brief Suicide Cognitions Scale (B-SCS), a simple and brief measure of suicide risk. The B-SCS provides a brief measure that captures critical aspects of suicide risk embedded in core beliefs about the self as unlovable, one's emotional experience as unbearable, and life problems as unsolvable (i.e., the suicidal belief system), resulting in chronic or enduring suicide risk and heightened vulnerability for acute episodes secondary to internal and external triggers. Data were analyzed from three diverse samples, including a student sample (*N* = 349), an inpatient psychiatric sample (*N* = 160), and a sample of emergency department (ED) patients presenting secondary to a suicidal crisis (*N* = 94). Those in the student and inpatient samples completed additional symptom measures (hopelessness, anxiety, depression) and the ED sample provided 6-month follow-up data for suicide attempts. Reliability (internal consistency, test-retest), concurrent validity, construct (divergent, convergent) validity, factorial, incremental, and predictive validity were evaluated, along with calculation of predictive value of negative and positive tests, sensitivity, and specificity estimates. The B-SCS demonstrated good reliability and validity, a unidimensional factor structure across samples, along with good predictive validity and value in real-world clinical settings. The B-SCS is a brief, reliable and valid measure of suicide risk, with good ability to identify those with enduring risk for subsequent suicide attempts. The B-SCS offers a unique contribution to understanding and assessing the nature of suicide risk over time targeting the suicidal belief system, with easy application across inpatient and outpatient clinical settings, and good predictive value.

## Introduction

Recent findings have highlighted the limited predictive value of traditional approaches to assessing suicidal thinking and behaviors in real-world clinical settings, specifically the limitations of relying on endorsement of suicidal thinking. Both Bjureberg et al. (1) and Simpson et al. (2), utilizing large samples from psychiatric emergency departments with suicide outcomes at 30 and 365-days post-discharge, found the Columbia Suicide Severity Rating Scale (C-SSRS) screener to have poor predictive value. Similarly, (3) found that prior suicidal thoughts and behaviors only provided “marginal improvement in diagnostic accuracy above chance (p. 2).” In the largest meta-analysis to date, covering a span of 50 years, (4) similarly found: (a) that predictive value has not improved, (b) predictive values are only slightly better than chance across all outcome measures, (c) and ultimately called for fundamental changes in approach to suicide risk assessment, particularly across real-world clinical settings.

The application of ecological momentary assessment (EMA) to suicidal thinking has, arguably, revealed one possible reason for the poor predictive value of traditional approaches. More specifically, EMA studies [e.g., (5, 6)] have revealed significant natural variability in suicidal thinking, negative affect, and individual history of self-harm, with fluctuations on an hour to hour, day to day, and week to week basis, characterized by wide shifts for those at highest risk. EMA also revealed periods of suicidal thinking that are likely undetected by traditional, retrospective approaches. Fluid vulnerability theory [FVT; (7)], a theory addressing the variable nature of suicide risk over time, offers a fundamental distinction between chronic and acute suicide risk, and an approach to understanding the rapid shifts in suicide risk revealed by EMA. Acute episodes of risk are believed to be time-limited and driven predominantly by situational and contextual variables (e.g., severity and mix of current symptoms, life stressors, substance abuse, nature of suicidal thinking, access to method), while chronic suicide risk represents enduring individual vulnerability for suicidality that cuts across multiple domains, including cognitive, physiological, affective, motivational, and behavioral. Ultimately, chronic or enduring suicide risk describes an individual's “ baseline” level of risk, or their underlying vulnerability to experience an acute episode in the future, an idea that is not markedly dissimilar from what Maris (8) originally referred to as “suicidal careers.” In short, FVT argues suicide risk fluctuates over time and when an acute episode of risk resolves (e.g., following a suicide attempt), the individual returns to a unique vulnerability “set point,” their baseline level of risk or what can be referred to as “residual risk,” a level determined by their life experiences (e.g., previous trauma, abuse, genetic history), biology/physiology, cognitive, affective, motivational makeup, and behavioral history.

Approaches to assess and understand suicidality have disproportionately focused on measuring acute risk episodes, and frequently take the form of assessing suicidal ideation, plans, intent, and urges, with predictive shortcomings summarized above. By comparison, efforts to assess and understand underlying and enduring individual vulnerability to suicidality, or chronic risk, have been more limited. The construct of hopelessness has played a central role in understanding suicide risk over time [e.g., (9, 10)], cutting across both acute and chronic risk dimensions. As originally conceptualized by Abramson et al. (11) hopelessness had both enduring features (i.e., trait) and contextual ones (i.e., state), noting that trait hopelessness involved negative future expectancies that cut across most domains of daily functioning, driven in large part by core beliefs about self, with core beliefs that are self-punitive, negative, and derogatory in nature being especially key. As a result, hopelessness has often been a key variable in understanding and assessing enduring risk [e.g., (9)]. Similarly, the constructs of perceived burdensomeness [e.g., (12)] and acquired capability (13) have both been demonstrated to be critical to understanding and capturing enduring suicide risk, as discussed in Joiner's (14) Interpersonal-Psychological Theory of Suicide. In FVT, core beliefs about self are referred to as the “suicidal belief system” and are characterized by pervasive identity-based hopelessness and captured, to some degree, by core beliefs about the self as unlovable, one's emotional experience as unbearable, and one's life problems as unsolvable.

Similarly, Wenzel et al. (15) emphasized the importance of “schemas” in understanding suicide risk and related states over time. In particular, they emphasized the importance of trait hopelessness and “unbearability,” with both representing enduring vulnerability to suicidal episodes, consistent with the notion of chronic risk in FVT. The idea of enduring vulnerability and subsequent emergence of an acute suicidal episode was also captured by the suicidal mode (16), an elaboration of traditional linear cognitive theory and models (17), including cognitive, behavioral, affective and motivational dimensions. Enduring vulnerability to new episodes of suicidality following resolution of an acute episode of suicidality represent “residual risk,” with the suicidal belief system and trait hopelessness being a critical component, and capturing much of its explanatory power (7).

The original Suicide Cognitions Scale [SCS; Rudd et al.^1^] was developed to measure suicide-specific and identity-based hopelessness consistent with FVT and the suicidal mode (7). SCS items are believed to tap the source of suicidal hopelessness along two primary themes, that is, two dimensions of the cognitive triad: self and others (18). Within the self-theme, items address sources of hopelessness well-documented in the suicide literature including unlovability (18, 19), helplessness [e.g., (20, 21)], and poor distress tolerance (22), the latter of which has been referred to as unbearability by Wenzel et al. (15). Within the other-theme items assess the construct of perceived burdensomeness (23, 24).

Subsequent work on the SCS has been promising, with findings across settings and populations suggesting three potential factors that have been termed unlovability, unbearability, and unsolvability (25–28), consistent with the original conceptualization of the suicide belief system and FVT (16). Ellis and Ruffino (28) also found evidence to support the idea that the SCS measured enduring suicide risk consistent with identity-based hopelessness and core beliefs that the self was unlovable, one's emotional experience was unbearable, and one's life problems were unsolvable. Subsequent research using bifactor modeling indicates that although the SCS has some multidimensionality, as theorized and demonstrated, item responses are also strongly influenced by a general latent factor (29). These findings suggest that that unlovability, unbearability, and unsolvability are influenced by a common, underlying suicidal belief system captured by the items in the Brief Suicide Cognitions Scale.

Accumulating research also supports the SCS's validity as an indicator of emerging and residual suicide risk. For instance, SCS scores significantly differentiate those who have attempted suicide from those with a history of suicide ideation only, and prospectively predict suicide attempts even when accounting for suicidal ideation (25, 29). In light of these findings, researchers have tested and validated shortened versions of the scale that may be more practical for use in clinical settings as a screening and/or assessment tool (26, 29). Taken together, these studies suggest the SCS assesses aspects of suicidality that are distinct from suicidal ideation, plans, intent and urges, consistent with recent calls for new approaches (30), and arguably the most important contribution of the B-SCS.

As evidence has emerged to support the SCS and its ability to assess unlovability, unbearability, and unsolvability and its unique role in understanding and targeting suicide risk over time, questions persist about the construct of identity-based hopelessness and how best to capture: (a) chronic or enduring suicide risk consistent with FVT, (b) the suicidal belief system embedded within the suicidal mode, and (c) the idea of residual risk following an acute episode (7). Over the course of the last several years, FVT has been modified to include a more refined and precise understanding of identity-based hopelessness specific to the issue of suicidality, noting that identity-based hopelessness is captured by the constructs of unlovability, unbearability, and unsolvability. The B-SCS captures suicide risk embedded in beliefs about the self as unlovable, one's emotional experience as unbearable, and life problems as unsolvable (i.e., the suicidal belief system), resulting in chronic suicide risk or heightened vulnerability for the emergence of acute suicidal crises over time and elevated residual risk after an acute episode has resolved. Consistent with FVT, unlovability, unbearability, and unsolvability are what creates enduring vulnerability (i.e., chronic risk) and greater likelihood that an acute episode of suicidality will be triggered in some individuals, along with the idea of “residual risk” following an attempt or acute episode of suicidality. A review of the SCS item content and previous findings convinced us it was possible to capture risk across the suicide belief system in a simple, brief, and potentially unidimensional scale (i.e., the general latent factor noted above), representing a natural evolution of the suicidal belief system and, hopefully one that is: (a) easier to use in clinical settings, (b) effective in predicting suicide risk and behavior over time, and (c) helpful in the treatment of specific aspects of underlying identity beliefs that generate enduring individual vulnerability. Consistent with these objectives, the current study included the following aims:

1. Development of the B-SCS that would be easy to use in real-world clinical settings.
2. Development of a brief measure that predicts suicidal behavior.
3. Exploration of the reliability and validity, including construct, factorial, incremental, and predictive validity, of the B-SCS.
4. Initial exploration of scale properties across both clinical and non-clinical samples.
5. Review of possible clinical applications of the scale, including the assessment and treatment of suicide risk.

## Methods

### Participants

Participants included three diverse samples, a non-clinical sample of undergraduate students (*N* = 349) participating in research as a requirement for an introductory psychology course, a clinical sample of consecutive inpatient admissions to a psychiatric unit in a major tertiary care medical center (over an 18-month period; *N* = 160), and a clinical sample of 94 suicide attempters (*N* = 53) and ideators (*N* = 41) presenting to an emergency department in a tertiary care medical center. The three studies did not occur simultaneously, rather were conducted over approximately a 24-month time frame. There were no exclusion criteria for the undergraduate sample, aside from evidence of an acute crisis warranting emergency evaluation and intervention (i.e., individual distress or upset during the study). No students were excluded for this reason, however. Exclusion criteria for the two clinical samples included: (a) active psychosis, (b) cognitive impairment that prevented participation (e.g., dementia, delirium, diminished intellectual functioning), and (c) impairment in reading and comprehension that resulted in an inability to read, understand, and complete study instruments, as well as the informed consent documents (e.g., severe learning disorders). As the study was reviewed and approved by the institutional review board, all participants reviewed and signed appropriate informed consent documents. Each sample is described in more detail below.

#### Sample 1: Undergraduate Students

The student sample included 291 females (83%) and 58 males (17%) participating in research for extra credit for an introductory psychology course, for a total *N* of 349. Ages ranged from 18 to 33, with a mean of 19. There was reasonable self-identified diversity in the sample, with 34 African-American (10%) participants, 29 Hispanic/Latino (8%), 31 Asian (9%), 1 Native American (0.3%), 1 East Indian (0.3%), 247 White/Non-Hispanic (71%), and 6 participants designated as “other” (1.4%). With respect to marital status, the overwhelming majority were single and had never been married (*N* = 345, 99%), with only 4 (1%) reporting being married. In terms of academic status, the majority were freshman (*N* = 165, 47%), with 87 (25%) sophomores, 61 (18%) juniors, and 36 (10%) seniors. Although clinical diagnostic assessments were not completed on the student sample, participants did complete a few additional demographic and personal history questions, including one on previous mental health care. Additionally, the full Beck Scale for Suicidal Ideation (31) was administered, and item 20 on the scale allowed us to identify those with a previous history of suicide attempts. As would be expected with a non-clinical student sample, a relatively small number, 40 (11%), reported a previous history of mental health care (either inpatient or outpatient) and a similar number (31, 9%) reported a previous history of at least one suicide attempt, providing limited variability in the sample and the opportunity for clinical comparisons.

#### Sample 2: Psychiatric Inpatients

The first clinical sample included 160 consecutive admissions to an inpatient psychiatric unit, including 113 females (71%) and 47 males (29%). The mean age was 40, with a range of 18–81 years old. As with the student sample, self-identified diversity was reasonable with 16 (10%) African-American participants, 3 (2%) Native American, 17 (11%) Hispanic/Latino, 122 (76%) White/Non-Hispanic participants and two indicating ethnicity as “other” (1%). In terms of marital status, 38 (24%) were single and never married, 60 (38%) married, 18 (11%) separated, 34 (21%) divorced, and 10 (6%) widowed. With respect to educational history, 22 (14%) reported that they did not have a high school degree, 81 (51%) graduated from high school, 26 (16%) attended some college, 21 (13%) completed a college degree, and 10 (6%) had advanced degrees.

Participants in this clinical sample were administered a brief demographic interview with a trained nurse to obtain background information (ethnicity, educ1ation level, marital status, number of previous psychiatric hospitalizations if any, and number of previous suicide attempts if any). Suicide attempt status and Axis-I clinical diagnosis(es) were determined by reviewing the admission note in the medical record. Not all subjects received an Axis I diagnosis in the chart, with some receiving a deferred diagnosis and others receiving a primary diagnosis on Axis II. Fifty-eight (36%) participants did not receive an Axis I diagnosis, with indications that the diagnosis was pending or the primary diagnosis was on Axis II. After completion of the history and chart review, participants completed all self-report measures in random order. As part of the participant's discharge paperwork, they were also given the B-SCS items. The length of stay for the clinical sample ranged from 1 to 21 days, with a mean of 4.8 days.

The most common primary Axis I diagnosis was a depressive disorder (including major depressive disorder both single and recurrent episodes, along with depressive disorder not otherwise specified), with 60 (38%) subjects diagnosed as depressed, followed by substance abuse (*N* = 18, 11%) (including alcohol, cannabis, opioids, and polysubstance abuse), bipolar disorder (*N* = 9, 6%) and adjustment disorder (*N* = 5, 3%). The most frequent Axis II was borderline personality disorder, with 19 (12%) participants receiving this diagnosis. A total of 100 (63%) participants were admitted for a suicidal crisis and 68 (43%) reported multiple admissions over their lifetime, with a range of 1–25 and a mean of two. Ninety (56%) reported that it was their first admission for suicidality. A total of 87 (54%) participants reported a prior history of suicide attempts regardless of the status of the current admission, with a range of 1–20, and a mean of 2.5 previous suicide attempts, providing an opportunity to compare ideators, single attempters, and multiple attempters. As mentioned above, suicide attempt status at the time of admission was accomplished by clinical chart review, with the admitting psychiatrist indicating suicide intent and categorizing the behavior as a suicide attempt. Participants completed the Beck Depression Inventory [BDI; (32)], the Beck Hopelessness Scale [BHS; (33)], the Beck Anxiety Inventory (34), the Beck Scale for Suicide Ideation (35), and the Trait Suicidality Scale items.

#### Sample 3: Emergency Department Patients

The second clinical sample included a total of 94 participants (51 making suicide attempts, 43 experiencing suicidal thoughts) presenting to an emergency department at a tertiary care medical center during an acute episode of suicidality. A significant majority of the sample (*N* = 77, 81.9%) reported previous episodes of suicidality. The mean age was 38 years (*SD* = 10.5), and a range of 18–58 years, with 51 females and 43 males. Self-identified diversity was reasonable with 58 (61.7%) reporting as White, 22 (23.4%) Black, 12 (12.8%) Hispanic, and 2 (2.1%) Native American. With respect to marital status, 32 (34%) reported being single/never married, 23 (24.5%) married, 19 (20.2%) divorced, 7 (7.4%) separated, 9 (9.6%) cohabitating, and 4 (4.3%) widowed. Although additional measures were not collected, participants agreed to a 6-month phone follow-up call to assess the presence of any subsequent suicide attempts (i.e., suicide attempt Time 2), allowing an opportunity to address the predictive value of the B-SCS items. A total of 16 suicide attempts were reported at T2 (17% of the original sample).

### Measures

All participants in Samples 1 and 2 completed the same measurement battery, with the order of presentation randomized, those in Sample 3 did not, they completed the B-SCS items and a 6-month follow-up call to assess the presence of any subsequent suicide attempts (i.e., suicide attempt Time 2). Total completion time varied across subjects, with a range of ~20–60 min. As mentioned above, Sample 2 participants completed the B-SCS items at both admission and discharge and Sample 3 had a 6-month follow-up interview regarding subsequent suicide attempts.

#### Beck Hopelessness Scale

Beck Hopelessness Scale [BHS; (33)]. The Beck Hopelessness Scale (BHS) is a 20-item, true-false scale that was designed to measure the degree to which a person's cognitions are dominated by negative future expectancies (36). Scores are obtained by summing the keyed responses which yields a score between 0 and 20. The BHS has been found to have high internal consistency reliability (K-R 20s typically in the 0.90's) in previous research (9) and good validity. Correlations for the BHS with clinical ratings of hopelessness are in the 0.70's (33). In the present study KR-20 was 0.94 for the clinical sample and 0.96 for the student sample.

#### Beck Depression Inventory-II

Beck Depression Inventory-II [BDI; (32)]. The Beck Depression Inventory-II (BDI-II) is a 21-item, self-report scale that has been used widely in research. Scores are obtained by summing the 21 ratings yielding a score that ranges from 0 to 63. The BDI has accrued a sound research base (37) with sound psychometric properties. High levels of convergent and divergent validity, as well as reliability have been established in prior research (37, 38). In the present study, internal consistency reliability was good with a Cronbach's alpha of 0.93 for the clinical sample and 0.87 for the student sample.

#### Beck Anxiety Inventory

Beck Anxiety Inventory [BAI; (33)]. The Beck Anxiety Inventory (BAI) is a 21-item self-report scale that was designed to measure the breadth and intensity of anxiety symptoms. BAI scores are calculated by summing the item scores and yields a score between 0 and 63. The BAI has shown high internal consistency and convergent validity (34). In the present study, Cronbach's alpha was 0.93 for the clinical sample and 0.97 for the student sample.

#### Beck Scale for Suicide Ideation

Beck Scale for Suicide Ideation [BSS; (31)]. The Beck Scale for Suicide Ideation is a 21-item self-report scale that was designed to measure suicidal ideation. BSS scores are calculated by summing item scores and yields a score between 0 and 63. The BSS has been shown to possess high internal consistency and convergent validity, with a previous alpha of 0.90 (35). In the present study Cronbach's alpha was 0.95 for the clinical sample and 0.81 for the student sample.

#### Brief Suicide Cognitions Scale

Brief Suicide Cognitions Scale (B-SCS). The B-SCS is a 6-item self-report scale that was designed to measure the suicidal belief system using items originally developed for the SCS, capturing enduring or identity-based hopelessness embedded in core beliefs about the self as unlovable, one's emotional experience as unbearable, and one's life problems as unsolvable (i.e., the elements of the suicidal belief system), resulting in persistent vulnerability for the emergence of acute suicidal crises over time secondary to both internal and external triggers. The six items selected for the B-SCS include two items each for unlovability, unbearability, and unsolvability, and were those that evidenced the strongest factor loadings across previous research, coupled with content validity. B-SCS employs Likert-scaling (1–5) and scores are calculated by summing the keyed responses and yields a score between 6 and 30. Only on item mentions the word “suicide.” Reliability and validity data are presented below.

## Results

### Internal Consistency and Test-Retest Reliability

Internal consistency reliability for the B-SCS was excellent, particularly for a six-item scale, with Cronbach's alpha of 0.90 for Sample 1 (students), 0.91 for Sample 2 (psychiatric inpatients), and 0.84 for Sample 3 (ED patients). A small group from Sample 1 (*N* = 33) was randomly selected to test the general stability of scores over a very brief period of time, i.e., 5 days. The test-retest reliability coefficient (Pearson product-moment correlation) for the initial B-SCS and that taken 5 days later was 0.84 (*p* < 0.0001), consistent with expectations that scores would remain reasonably stable in non-clinical samples. As mentioned above, Sample 2 was tested at both intake and discharge (i.e., a second B-SCS was administered at discharge). Unlike Sample 1, however, it was anticipated that there would be some change in B-SCS scores from admission to discharge, consistent with symptom recovery and stabilization during the course of the hospital stay for an acute episode of suicidality. However, we did anticipate continued clinical elevation in scores and greater temporal stability given the enduring nature of core beliefs and the suicidal belief system. This was evident in the test-retest reliability coefficient (Pearson product-moment correlation) of 0.46 (*p* < 0.0001), with a moderate value, again consistent with the nature of the construct.

### Item Analysis and Content Validity

The means, standard deviations, item-total correlations, and item-level responses are provided in Table 1. Item-level responses mirror what would be anticipated across all three samples, including marked elevations in item-level scores at higher levels (i.e., 4-agree or 5-strongly agree) for the two clinical samples in contrast to the non-clinical student sample. Across all three samples, the item-total correlations also suggested appropriate item inclusion, with all correlations significant beyond the 0.001 level.

**Table 1:** Item descriptive statistics for B-SCS.

**Item**	***M***	***SD***	Item-total correlation^*^	**% Endorsing each response option**				
				**1**	**2**	**3**	**4**	**5**
**Inpatient sample (** ***N*** **=** **160)**								
I am completely unworthy of love.	2.75	1.45	0.72	26.8	23.5	14.4	19.0	16.3
Nothing can help me solve my problems.	2.59	1.32	0.77	25.2	29.0	17.4	18.1	10.3
I can't cope with my problems any longer.	3.43	1.33	0.79	12.3	12.9	20.6	27.7	26.5
I can't imagine anyone being able to withstand this kind of pain.	3.45	1.41	0.71	14.8	11.0	18.7	25.2	30.3
There is nothing redeeming about me.	2.77	1.33	0.78	22.8	20.8	25.5	18.1	12.8
Suicide is the only way to end this pain.	2.45	1.35	0.75	34.2	20.0	22.6	12.9	10.3
**ED patient sample (** ***N*** **=** **94)**								
I am completely unworthy of love.	2.44	1.31	0.65	27.7	36.2	10.6	16.0	9.6
Nothing can help me solve my problems.	2.33	1.22	0.52	26.6	42.6	9.6	13.8	7.4
I can't cope with my problems any longer.	3.12	1.44	0.71	17.0	25.5	7.4	28.7	21.3
I can't imagine anyone being able to withstand this kind of pain.	3.45	1.33	0.52	8.5	24.5	6.4	35.1	25.5
There is nothing redeeming about me.	2.51	1.28	0.65	24.5	36.2	10.6	21.3	7.4
Suicide is the only way to end this pain.	2.51	1.47	0.68	33.0	27.7	10.6	12.8	16.0
**Student sample (** ***N*** **=** **349)**								
I am completely unworthy of love.	1.25	0.72	0.70	86.2	7.1	3.0	3.0	0.7
Nothing can help me solve my problems.	1.25	0.61	0.77	81.7	13.1	3.7	1.1	0.4
I can't cope with my problems any longer.	1.23	0.63	0.76	85.1	9.0	4.1	1.5	0.4
I can't imagine anyone being able to withstand this kind of pain.	1.27	0.75	0.68	85.1	7.5	3.7	2.6	1.1
There is nothing redeeming about me.	1.21	0.57	0.82	84.8	10.8	3.3	0.7	0.4
Suicide is the only way to end this pain.	1.07	0.35	0.60	95.1	3.7	0.7	0.0	0.4

* *All item-total correlations significant a p < 0.0001*.

### Concurrent Validity

Table 2 provides the correlation matrix for all study variables for Sample 1 and Sample 2. As noted, participants in Sample 3 did not complete the assessment battery. The table includes the admission and discharge administrations for the B-SCS (B-SCS-A for admission and B-SCS-D for discharge); as indicated, these only apply to Sample 2. In order to address any concern about construct overlap, a revised B-SCS score was calculated, deleting the one B-SCS item (#6) that used the word “suicide” in item content, thereby allowing comparisons uncompromised by any conceptual overlap due to item content.

**Table 2:** Correlations of B-SCS with other clinical variables across inpatient and student samples.

	**BDI**	**BHS**	**BSS**	**BAI**	**B-SCS-A**
**Inpatient sample (** ***N*** **=** **160)**					
B-SCS-admission	0.71^**^	0.75^**^	0.72^**^	0.35^**^	
B-SCS-discharge	0.33^**^	0.47^**^	0.39^**^	0.14 NS	0.46^**^
B-SCS-revised (B-SCS-discharge scores)	0.31^**^	0.45^**^	0.32^**^	0.12 NS	
**Student sample (** ***N*** **=** **349)**					
B-SCS	0.34^**^	0.13^*^	0.32^**^	0.08 NS	

** *Correlation is significant at the 0.01 level*.

* *Correlation is significant at the 0.05 level*.

*NS, non-significant; BDI, Beck Depression Inventory-II; BHS, Beck Hopelessness Scale; BSS, Beck Scale for Suicide Ideation; BAI, Beck Anxiety Inventory*.

As expected, correlations between the B-SCS and measures of hopelessness, suicidal ideation, depression, and anxiety (see Table 2) were significant. Correlations were significant across all samples and, appropriately, the strongest relationships were evidenced in the clinical samples. Also as expected, the strongest relationships were with measures of depression, suicidal ideation, and hopelessness, all of which are critical in the assessment, management, and treatment of suicidality (24). B-SCS-revised correlations were also significant, evidencing the same pattern as the B-SCS across both samples and no apparent distortion secondary to item overlap.

### Construct Validity: Convergent and Divergent Aspects

Given the stated purpose of the B-SCS to assess trait suicidality, the scale should effectively differentiate high-risk groups from others in the general population, suicidal and non-suicidal clinical patients, and those with a range of suicide risk in clinical populations. In Sample 1, B-SCS scores were significantly higher for those with a past history of self-reported suicide attempts (*M* = 9.8, *SD* = 4.1) in comparison to those with no suicidal history (*M* = 7.1, *SD* = 2.9) (*F* = 7.57, *p* = 0.006; Cohen's *d* = 0.76). Similarly, B-SCS scores were significantly lower for those in Sample 1 with no previous history of mental health treatment (*M* = 7.1, *SD* = 2.9) when compared to those with a treatment history (*M* = 8.5, *SD* = 4.0) (*F* = 4.16, *p* = 0.043, Cohen's *d* = −0.40). In Sample 2, the B-SCS at admission effectively discriminated between those with a past history of suicide attempts (*M* = 19.0, *SD* = 6.7) vs. those without (*M* = 15.7, *SD* = 6.5) (*F* = 8.23, *p* = 0.005, Cohen's *d* = 0.50). This finding was similar at discharge, with significantly higher B-SCS scores among those with a history of suicide attempts (*M* = 14.1, *SD* = 6.28) when compared to those with no history of attempts (*M* = 11.1, *SD* = 4.52) (*F* = 9.33, *p* = 0.003, Cohen's *d* = 0.55). Also consistent with the extant literature and hypotheses, there were significant differences in B-SCS scores across diagnostic groups, with the those diagnosed with a bipolar disorder or major depression (*M* = 13.87, *SD* = 5.67) evidencing significantly higher scores in contrast to those with a primary diagnosis of an anxiety disorder (*M* = 11.2, *SD* = 4.88) (*F* = 5.77, *p* = 0.018; Cohen's *d* = 0.51). Among the measures used, the BAI is that with the weakest relationship to suicidality (31). Finally, as hypothesized, the B-SCS differentiated between multiple attempters (*N* = 50) and ideators (*N* = 66) (*F* = 14.32, *p* = 0.0001, Cohen's *d* = 0.77), and multiple attempters (*N* = 50) and single attempters (*N* = 30) (*F* = 6.08, *p* = 0.016, Cohen's *d* = *0.6*0) in Sample 2, consistent with previous findings regarding significantly higher chronic risk among multiple attempters (39). Findings regarding past history of suicide attempt, previous history of mental health treatment, and diagnosis were comparable when B-SCS item #6, that used the word “suicide,” was deleted.

Given that the B-SCS is capturing enduring suicide risk in a manner different from traditional approaches like hopelessness, potential unique explanatory power beyond the traditional BHS was explored. In short, if the B-SCS offers unique explanatory power, it should evidence incremental validity when compared with the BHS. In the student sample, the partial correlation between the BSS and B-SCS, controlling for the BHS was 0.33 (*p* = 0.0001) clearly consistent with good incremental validity. In contrast, the partial correlation between the BSS and BHS, controlling for the B-SCS was 0.09 (*p* = 0.251), with the BHS adding no significant explanatory power beyond that accounted for by the B-SCS. Results were similar in Sample 2, with the partial correlation between the BSS and B-SCS, controlling for hopelessness, of 0.45 (*p* = 0.0001), with the B-SCS adding incremental explanatory power. Again, results were comparable when B-SCS item #6, that used the word “suicide” was deleted partial correlation between the BSS and B-SCS, controlling for hopelessness was 0.39 (*p* = 0.0001).

### Predictive Validity and Residual Risk

In Sample 3 participants agreed to a 6-month follow-up call to assess the occurrence of any subsequent suicide attempts, allowing an opportunity to address the predictive value of the B-SCS, including the idea that trait suicidality results in heightened “residual risk” following resolution of and acute episode of suicidality. A total of 16 suicide attempts were reported during follow-up (17% of the original sample). Mean B-SCS scores were significantly higher for those making a follow-up suicide attempt (*M* = 19.7, *SD* = 4.2), in comparison to those without (*M* = 15.2, *SD* = 6.4) (*F* = 7.21, *p* = 0.008; Cohen's *d* = 0.83).

**Figure 1:**
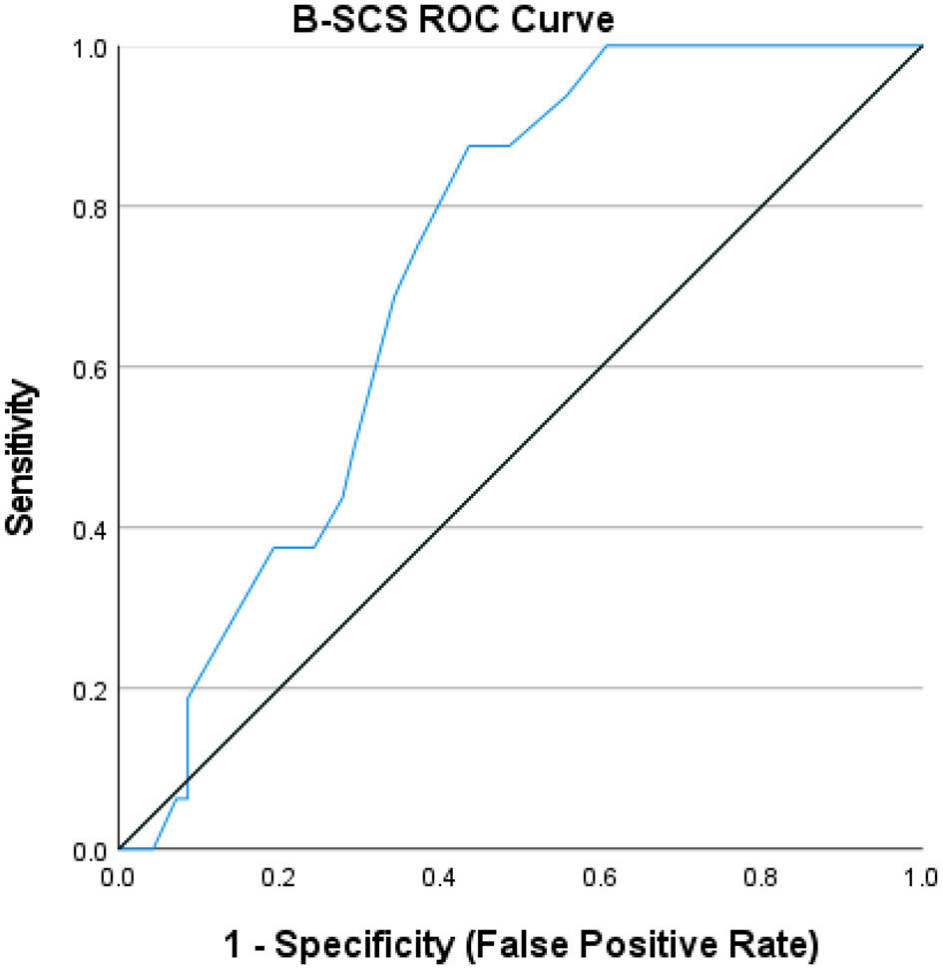
B-SCS ROC Curve.

The positive predictive value, negative predictive value, sensitivity and specificity of the B-SCS were calculated next. As previously illustrated in Table 1, the item-level responses for the B-SCS demonstrate the remarkably low frequency nature of endorsement outside of the 1–2 range (i.e., 2-disagree or 1-strongly disagree), particularly in a non-clinical sample. Given the nature of item content, endorsement distributions, and the purpose of the B-SCS (to identify enduring risk), the cutoff score was set at a score of ≥13 (approximately one SD below the mean for those admitted to an inpatient unit). Subsequent analysis revealed that the B-SCS was able to uniformly identify those at risk for a follow-up suicide attempt (χ2 = 7.77, *p* = 0.005), with negative predictive value of a test of 1.00, positive predictive value of 0.230, sensitivity of 1.00, and specificity of 0.346. The lower specificity is not particularly surprising given the nature of the sample, that is, those presenting at the emergency department in suicidal crisis (See Table 3). The receiver operating characteristics curve is presented in Figure 1, with the area under the curve (AUC) of 0.724, falling in the adequate range, not surprising given both the small sample size and limited number of subsequent attempts (*N* = 16, 17% of original sample).

**Table 3:** B-SCS predictive value.

	**Follow-up suicide attempt**	**No follow-up suicide attempt**	**Totals**
B-SCS above cutoff 13	**16**	**51**	**67**
B-SCS below cutoff 13	**0**	**27**	**27**
Totals	**16**	**78**	**94**
Negative predictive value	1.00		
Positive predictive value	0.239		
Sensitivity	1.00		
Specificity	0.346		

*Bold values indicate number of participants reporting either suicide attempts or no suicide attempts at follow-up*.

### Factorial Validity

As mentioned previously, the B-SCS is conceptualized as a brief measure of enduring suicide risk, defined as identity-based hopelessness embedded in core beliefs about the self as unlovable, one's emotional experience as unbearable (unbearability), and one's life problems as unsolvable (unsolvability), resulting in persistent vulnerability for the emergence of acute suicidal crises and residual risk after resolution of an acute episode. Given the exploratory nature of this initial study, principal components analysis was used, along with Cattell's (40) scree test to determine factor extraction based on the magnitude of the eigenvalues (cutoff set at >1) derived with the two clinical samples and the non-clinical student sample. Table 4 provides factor loadings for all items, with the scale proving unidimensional across all three samples. In Sample 1, a single factor accounted for 66% of the variance (eigenvalue of 3.96). In Sample 2, a single factor accounted for 69.3% of the variance (eigenvalue of 4.157) and in Sample 3, a single factor accounted for 56% of the variance (eigenvalue of 3.36). Current results support the construct of a general unidimensional measure of the suicidal belief system, with a comparable factor structure across both clinical and non-clinical samples.

**Table 4:** Item-factor loadings across samples.

**Inpatient sample (*N* = 160)**	**Item-factor loading**
I am completely unworthy of love.	0.81
Nothing can help me solve my problems.	0.84
I can't cope with my problems any longer.	0.86
I can't imagine anyone being able to withstand this kind of pain.	0.80
There is nothing redeeming about me.	0.85
Suicide is the only way to end this pain.	0.83
**ED patient sample (** ***N*** **=** **94)**	
I am completely unworthy of love.	0.79
Nothing can help me solve my problems.	0.74
I can't cope with my problems any longer.	0.84
I can't imagine anyone being able to withstand this kind of pain.	0.69
There is nothing redeeming about me.	0.79
Suicide is the only way to end this pain.	0.82
**Student sample (** ***N*** **=** **349)**	
I am completely unworthy of love.	0.80
Nothing can help me solve my problems.	0.85
I can't cope with my problems any longer.	0.84
I can't imagine anyone being able to withstand this kind of pain.	0.78
There is nothing redeeming about me.	0.88
Suicide is the only way to end this pain.	0.72
**Sample for CFA (** ***N*** **=** **160)**	
I am completely unworthy of love.	0.82
Nothing can help me solve my problems.	0.78
I can't cope with my problems any longer.	0.75
I can't imagine anyone being able to withstand this kind of pain.	0.73
There is nothing redeeming about me.	0.84
Suicide is the only way to end this pain.	0.72

To further confirm the unidimensionality of the B-SCS, a confirmatory factor analysis (CFA) was conducted in Sample 2 using the MPlus 7.4 software (41). To account for skewed item responses, we used a robust maximum likelihood estimator with all 6 items loading onto a single factor. Results indicated marginal fit [root mean square error of approximation (RMSEA) = 0.22, 95% confidence interval (CI) = 0.17–0.26; comparative fit index (CFI) = 0.84; standardized root mean square residual (SRMR) = 0.06]. Review of the modification indices suggested model fit could be improved if the following residuals were allowed to correlate, however: items 1 and 4, items 3 and 4, and items 2 and 6. When repeated with these additional parameters, model fit improved dramatically: RMSEA = 0.08, 95% CI = 0.00–0.15; CFI = 0.99; SRMR = 0.02. Item-factor loadings for this final model exceeded 0.70 (see Table 4).

## Discussion

The B-SCS performed well overall, across both non-clinical and clinical samples, particularly for a brief measure developed for the unique demands of clinical settings. Current results support the strong psychometric properties for this brief measure capturing enduring suicide risk embedded in beliefs about the self as unlovable, one's emotional experience as unbearable, and life problems as unsolvable (i.e., the suicidal belief system), resulting in chronic suicide risk or heightened vulnerability for the emergence of acute suicidal crises over time and elevated residual risk after an acute episode has resolved. Findings demonstrate strong reliability estimates, particularly for a six-item measure, good evidence of concurrent, convergent, incremental and divergent validity, along with good evidence of predictive validity, consistent with FVT and the idea of chronic and residual suicide risk. In terms of factorial validity, findings suggest the B-SCS is unidimensional (across both clinical and non-clinical samples) and represents the suicidal belief system as described in FVT, the suicidal mode (7, 16) and the cognitive model of suicide (15). Findings confirmed that the B-SCS offers good incremental validity, with unique predictive value when compared to frequently used measures of hopelessness, like the Beck Hopelessness Scale. In particular, the importance of recognizing, understanding, and assessing enduring or chronic suicide risk (i.e., the suicidal belief system including unlovability, unbearability, and unsolvability) as part of a comprehensive suicide risk assessment is supported.

As results revealed, the B-SCS faces the same clinical screening and predictive validity challenges as other measures in clinical settings, with an AUC of 0.724 and limited specificity, despite good sensitivity at a low cutoff score. Although additional study is certainly needed, particularly with larger samples and longer follow-up periods, results across both non-clinical and clinical samples suggest the B-SCS has potential as a brief screening measure targeting enduring individual vulnerability. The mere presence of unlovability, unbearability, and unsolvability can elevate risk and the B-SCS could potentially offer a response to the screening problems revealed by recent EMA findings and significant, natural variations in suicidal thinking over time.

Current findings have a number of significant implications for clinical practice, particularly suicide risk assessment, and treatment. As described in FVT (7) accurate assessment of suicide risk cuts across both acute and chronic domains. The assessment of acute risk and related contextual variables such as symptom severity, life stressors, suicidal thinking and intent is well chronicled [e.g., (42)], but the issue of recognizing, understanding and assessing chronic risk in a reliable and valid manner has received less attention. The B-SCS is a simple and brief instrument that can be completed in a matter of minutes, one that will provide meaningful clinical information about enduring suicide risk. Additionally, only a single B-SCS item mentions the word “suicide,” offering a unique approach to recognizing vulnerability to suicide risk not reliant on current suicidal thinking. The B-SCS helps the clinician recognize, track, and target the suicidal belief system in treatment and related identity-based hopelessness (43). Of specific importance, an elevated B-SCS score indicates heightened vulnerability for future episodes, alerting clinicians to more closely monitor individuals, recognizing that residual risk does not resolve in parallel to an acute episode and related symptoms, and does not always parallel active suicidal thinking.

The current study is not without significant limitations, however. It should be noted that although the two clinical samples good representation of those experiencing suicidal thoughts and those making suicide attempts, subsequent studies should include individuals from both outpatient and inpatient settings, larger numbers, and greater heterogeneity in past suicidal thoughts and behaviors. Additionally, longer follow-up periods are needed to further address predictive value and validity, which is particularly important for a measure of chronic risk. In particular, the number of follow-up suicide attempt is relatively small, limiting statistical power and conclusions based on the current findings. Furthermore, Sample 3 did not include comprehensive assessment of other clinical symptoms or psychiatric diagnoses. In light of our findings that B-SCS scores significantly differed across participants with and without mood disorders in Sample 2, additional research investigating how B-SCS responses and performance across different clinical subgroups is warranted. Despite these limitations, the B-SCS is a promising measure of chronic or residual suicide risk, with considerable potential in clinical settings, particularly given it can be administered in a few minutes.

## Data Availability Statement

The raw data supporting the conclusions of this article will be made available by the authors, without undue reservation.

## Ethics Statement

The studies involving human participants were reviewed and approved by Texas Tech University Institutional Review Board. The patients/participants provided their written informed consent to participate in this study.

## Author Contributions

MR conceptualized the original study, collected the data, conducted data analysis, along with writing, and editing the final manuscript. CB contributed to data analysis and interpretation, along with manuscript writing, and editing. All authors contributed to the article and approved the submitted version.

## Conflict of Interest

The authors declare that the research was conducted in the absence of any commercial or financial relationships that could be construed as a potential conflict of interest.

## Publisher's Note

All claims expressed in this article are solely those of the authors and do not necessarily represent those of their affiliated organizations, or those of the publisher, the editors and the reviewers. Any product that may be evaluated in this article, or claim that may be made by its manufacturer, is not guaranteed or endorsed by the publisher.

## Footnotes

^1^Rudd MD, Schmitz B, McClenen R, Joiner TE, Elkins G, Claassen C. *The Suicide Cognitions Scale: Measuring Suicide-Specific Cognitions* (2008). unpublished manuscript.
